# Construction of Stretching-Bending Sequential Pattern to Recognize Work Cycles for Earthmoving Excavator from Long Video Sequences

**DOI:** 10.3390/s21103427

**Published:** 2021-05-14

**Authors:** Yiguang Wu, Meizhen Wang, Xuejun Liu, Ziran Wang, Tianwu Ma, Yujia Xie, Xiuquan Li, Xing Wang

**Affiliations:** 1 Key Laboratory of Virtual Geographic Environment, Ministry of Education, Nanjing Normal University, Nanjing 210023, China; yiguangwu@njnu.edu.cn (Y.W.); liuxuejun@njnu.edu.cn (X.L.); wangzr@njnu.edu.cn (Z.W.); tianwuma@nnu.edu.cn (T.M.); 171301029@njnu.edu.cn (X.L.); 181301021@njnu.edu.cn (X.W.); 2 Jiangsu Center for Collaborative Innovation in Geographical Information Resource Development and Application, Nanjing 210023, China; 3 School of Geography, Nanjing Normal University, Nanjing 210023, China; 4 School of Information Engineering, Nanjing Normal University Taizhou College, Taizhou 225300, China; 5 College of Information Engineering, Nanjing University of Finance & Economics, Nanjing 210023, China; 9120181003@nufe.edu.cn

**Keywords:** earthmoving projects, earthmoving excavators, surveillance cameras, reality factors, deep learning, work cycle, atomic action, sequential pattern, long video sequences, computer vision

## Abstract

Counting the number of work cycles per unit of time of earthmoving excavators is essential in order to calculate their productivity in earthmoving projects. The existing methods based on computer vision (CV) find it difficult to recognize the work cycles of earthmoving excavators effectively in long video sequences. Even the most advanced sequential pattern-based approach finds recognition difficult because it has to discern many atomic actions with a similar visual appearance. In this paper, we combine atomic actions with a similar visual appearance to build a stretching–bending sequential pattern (SBSP) containing only “Stretching” and “Bending” atomic actions. These two atomic actions are recognized using a deep learning-based single-shot detector (SSD). The intersection over union (IOU) is used to associate atomic actions to recognize the work cycle. In addition, we consider the impact of reality factors (such as driver misoperation) on work cycle recognition, which has been neglected in existing studies. We propose to use the time required to transform “Stretching” to “Bending” in the work cycle to filter out abnormal work cycles caused by driver misoperation. A case study is used to evaluate the proposed method. The results show that SBSP can effectively recognize the work cycles of earthmoving excavators in real time in long video sequences and has the ability to calculate the productivity of earthmoving excavators accurately.

## 1. Introduction

Some of the most important aspects of construction are earthmoving projects, which account for about 20% of the total expenditure of a project [1]. For construction cost control, it is crucial to accurately assess the productivity of earthmoving excavators. Key to this assessment is knowing the number of work cycles performed per unit of time [2]. In general, the number of work cycles is obtained by manual recognition by the manager at the construction site. The manager first needs to discern the working status based on the posture of the earthmoving excavator. The posture of the excavator is also referred to as atomic actions in this paper. Then, the recognized atomic actions are associated as a working cycle. However, such manual tasks are time-consuming, costly, and error-prone [3]. In recent years, surveillance cameras have been widely used in construction site management. An increasing number of researchers are using surveillance cameras in combination with computer vision techniques to develop methods for the automatic recognition of earthmoving excavator work cycles [3,4,5,6]. Computer vision (CV) is able to represent human vision and manual reasoning processes more realistically [7]. Therefore, it has become a popular method for recognizing the work cycles of earthmoving excavators.

There are four basic types of methods for recognizing the work cycles of earthmoving excavators using computer vision technology: (1) human-assisted recognition, (2) pre-conditioned recognition, (3) recognition based on temporal sequences, and (4) recognition based on sequential patterns. Human-assisted recognition first detects earthmoving excavators using manually extracted image features, and then recognizes the activity state of the earthmoving excavator from consecutive frame images based on the change in the center of the mass or shape of the bounding box. Finally, it relies on a human-assisted approach to discriminate the work cycle. For example, Zou et al. [8] used parameters such as hue, saturation, and value in the image color space to recognize and track the earthmoving excavator. Then, the changing center-of-mass coordinates of the earthmoving excavator in consecutive images taken at constant time intervals were used as indicators of movement to determine the activity and idle status of the earthmoving excavator. Another example is the image differencing method, which is used to detect the shape change of the earthmoving excavator to determine its activity and idle status [9]. Human-assisted recognition is unable to recognize atomic actions, which results in the recognition of work cycles relying on manual discrimination. It can be seen from the existing studies that such methods can only identify the active/idle states.

Pre-conditioned recognition is used to pre-segment the long video sequences captured by surveillance cameras into short videos containing only one work cycle. Then, the atomic actions are recognized in a single short video. For example, Golparvar-Fard et al. [3] classified the atomic actions in the work cycle of an earthmoving excavator into four categories, “Digging”, “Dumping”, “Hauling”, and “Swinging”, then used a histogram of gradient orientation (HOG) and a support vector machine (SVM) to recognize the atomic actions in short videos. The advantage of this method is that it can recognize atomic actions in the work cycles, but the starting point and duration of each atomic action need to be predefined in order to facilitate the segmentation of long video sequences. Therefore, this method cannot recognize work cycles in long video sequences.

Recognition based on temporal sequences constructs a set of atomic actions in the work cycle according to the temporal sequence of the recognized atomic actions, where a set of temporal atomic actions is discriminated as one work cycle. For example, Roberts et al. [10] classified the atomic actions in the work cycle of an earthmoving excavator into four categories: “Load Bucket”, “Swing Bucket”, “Dump”, and “Idle”. Then, convolutional neural networks (CNNs) and hidden Markov models (HMMs) were used to recognize the atomic actions, and finally a set of atomic actions containing temporal sequences was constructed. Chen et al. [2] classified the atomic actions in the work cycle of an earthmoving excavator into three categories: “Digging”, “Swinging”, and “Loading”. Then, three deep learning methods, Faster R-CNN, SORT, and 3D ResNet, were used to recognize the atomic actions. Finally, two occurrences of the “Digging” atomic action were used as the condition for work cycle recognition. Compared with the previous methods, this third type of method is able to recognize not only the atomic actions in the work cycle of an earthmoving excavator, but also the work cycle in a long video sequence. However, the recognition of work cycles based on temporal sequences is not in line with the actual situation of the construction site. For example, when there is another abnormal atomic action, “Digging”, in the same work cycle due to driver misoperation, the method will incorrectly recognize one work cycle as two work cycles, which is an abnormal work cycle. The abnormal work cycle cannot be filtered out by relying only on the temporal sequences, which increases the error rate of work cycle recognition.

**Figure 1:**
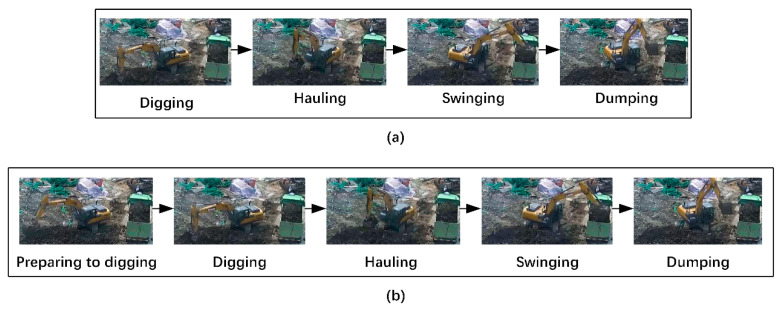
Atomic actions of an earthmoving excavator intercepted in a long video sequence: (**a**) four-atomic-action sequential pattern and (**b**) five-atomic-action sequential pattern.

Recognition based on sequential patterns associates the atomic actions in the work cycle with each other according to the operation sequence based on the actual work of the earthmoving excavator at the construction site to achieve recognition of the work cycle. For example, Kim et al. [11] classified the atomic actions in the work cycle of an earthmoving excavator into “Digging”, “Hauling”, “Dumping”, and “Swinging”, which they considered as following a fixed sequence of operations. This sequence is beneficial to filter out abnormal work cycles and achieve the correct recognition of work cycles. Therefore, they constructed the sequential pattern of “Digging→Hauling→Dumping→Swinging” (refer to Figure 1a, we call this the four atomic actions sequential pattern), then used convolutional neural networks and double-layer long short-term memory to model and train the visual features of the earth excavator and the sequential pattern of work cycles. The sequence pattern constructed by this method works according to the actual working conditions of earthmoving excavators in construction sites, and abnormal atomic actions caused by driver misoperation can be easily detected using the sequence pattern. For example, according to the sequence pattern, “Dumping” should be followed by “Swinging”. However, if “Digging” appears, it is easy to determine that “Digging” here is an abnormal atomic action. Constructing sequential patterns in this method is essential, but the sequential patterns [11] are not perfect at present. Moreover, the excessive and similar visual appearances of atomic actions are difficult to distinguish in videos, which increases the difficulty of recognition of atomic actions [11]. Besides, it was pointed out in the paper [2] that the double-layer long short-term memory used in this framework cannot recognize the work cycles of earthmoving excavators in long video sequences because of the gradient descent problem.

In summary, the first two methods are unable to automatically recognize the work cycles of earthmoving excavators in long video sequences. Compared with the first two types, the third one can recognize the work cycles of earth excavators in long video sequences, but still has some disadvantages, which means that using temporal sequences to recognize the work cycles according to the actual working conditions of earthmoving excavators in construction sites does not work. Two occurrences of the “Digging” atomic action are used as the condition for work cycle recognition, which makes this type of method inflexible and perform badly in filtering out abnormal atomic actions caused by driver misoperation in one work cycle. However, these abnormal atomic actions easily lead to abnormal work cycles and increase the recognition error rate of work cycles. According to our observation in the construction site, for example, the abnormal atomic actions generated by driver misoperation usually appear between “Dumping” and “Swinging”—that is, atomic actions similar to “Digging” appear again between the two, which makes it easy for one work cycle to be recognized as two work cycles.

The fourth type of method is the most advanced method at present. Its advancement is shown by the fact that the sequence pattern is constructed according to the actual working conditions of the earthmoving excavator in the construction site, which can filter out abnormal atomic actions and thus lead to a correct recognition of work cycles. However, two problems still exist in this method. The first problem is that the sequence pattern is not perfect, and as the key step of the method it is inappropriate to use “Digging” as the first atomic action of the work cycle. According to our observations in the construction site, the earthmoving excavator was not present in the current working area at the beginning, as it was being moved from other areas. The posture of the earthmoving excavator during the movement from other areas to the current work area was with the moving boom raised, the dipper handle perpendicular to the ground, and the bucket flattened at the end. When the excavator arrived at the current working area, the first atomic action in the work cycle was not “Digging”, but a preparatory action—i.e., “Preparing to dig”. The posture of this atomic action was such that the earthmoving excavator stretched the dipper handle and bucket to 80% of the maximum length of the arm, then lowered the boom and used the bucket to cut down the soil. This operation has the advantage of maximizing the digging power of the earthmoving excavator [12]. After adding the atomic action “Preparing to dig”, the four-atomic-action sequential pattern changed to a five-atomic-action sequential pattern (refer to Figure 1b)—i.e., “Preparing to dig→Digging→Hauling→Dumping→Swinging”. The second problem is that many atomic actions with a similar visual appearance make the atomic action recognition more difficult. For example, “Digging” and “Hauling” are similar, and “Preparing to dig”, “Dumping”, and “Swinging” are similar. “Dumping” and “Swinging” are similar. “Digging” and “Hauling” are similar in that the dipper handle is kept perpendicular to the ground and the bucket is parallel to the ground, but the difference is that when moving from “Digging” to “Hauling”, the boom needs to be raised. Therefore, it is difficult to clearly distinguish such small distinctions based on visual appearance. The similarities between “Preparing to dig”, “Dumping”, and “Swinging” are the stretching of the dipper handle and bucket. The difference is that during “Dumping” and “Swinging”, the boom needs to be raised. The nuances of these three atomic actions are also difficult to distinguish clearly by visual appearance. These two problems seem to be opposed to each other, and we need to add the number of atomic actions to improve the existing sequential pattern to make it more suitable for the actual situation of earthmoving excavators working in a construction site. However, the added atomic actions still have the problem of having a similar visual appearance to other atomic actions, which will further increase the difficulty of atomic action recognition.

The purpose of this paper is to construct a simplified sequential pattern by combining atomic actions with excessively similar visual appearances while taking into account the abnormal atomic actions caused by driver misoperation in order to correctly recognize the work cycles of earthmoving excavators in long video sequences. The details of the proposed method are presented in Section 2 of this paper. In order to verify the method, we conducted experiments using the surveillance video collected from an earthwork construction site and analyzed the experimental results, as detailed in Section 3. Section 4 contains the discussion. In the last section, we conclude the paper and describe directions for future work.

## 2. Method

### 2.1. Basic Idea and Overall Design

The sequential pattern constructed in this paper requires the consideration of two problems: the first problem is how to correctly distinguish between many atomic actions that are similar in visual appearance. The resolution of the first problem will enhance the precision of the atomic action recognition. The second problem is how to filter out the abnormal atomic actions caused by driver misoperation. The resolution of this problem will enhance the precision and robustness of the work cycle recognition.

The first problem is commonly solved by combining atomic actions with a similar visual appearance [10]. In this paper, we will combine “Preparing to dig”, “Dumping”, and “Swinging”, which have similar visual appearances, as well as the actions “Digging” and “Hauling”. When performing the “Preparing to dig”, “Dumping”, and “Swinging” atomic actions, the earthmoving excavator needs to stretch its arm to keep the bucket away from the boom, so these three atomic actions will be combined and named “Stretching”. During “Digging” and “Hauling”, the arm needs to be bent to keep the bucket close to the boom, so these two atomic actions will be combined and named “Bending”. Once the atomic actions with similar visual appearances are combined, the work cycle only consists of two atomic actions, “Stretching” and “Bending”, which have obvious visual appearance differences. A “Stretching→Bending” sequential pattern (SBSP) will be constructed.

For the second problem, the literature [11] filters out abnormal atomic actions according to the operation sequence of the atomic actions based on the sequential model. The abnormal atomic actions in the literature [11] are not the actual atomic actions in the work cycle, but are caused by the misrecognition of its atomic action recognition module, and do not take into account driver misoperation, which generates actually abnormal atomic actions. According to our observations at the construction site, work cycles composed of such abnormal atomic actions usually have a short duration, and will need to be filtered out by using the average completion time of the abnormal work cycle.

**Figure 2:**
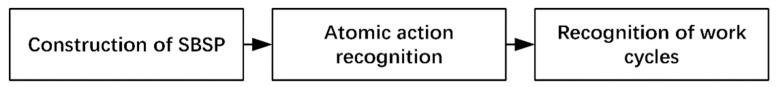
The framework of the proposed method based on stretching-bending sequential pattern (SBSP).

Based on the above basic ideas, the overall design of our method consists of three parts. The first part is the construction of the SBSP, the second part is the atomic action recognition, and the third part is the work cycle recognition. The overall design of our method is shown in Figure 2.

### 2.2. Construction of Stretching–Bending Sequential Pattern (SBSP)

**Figure 3:**
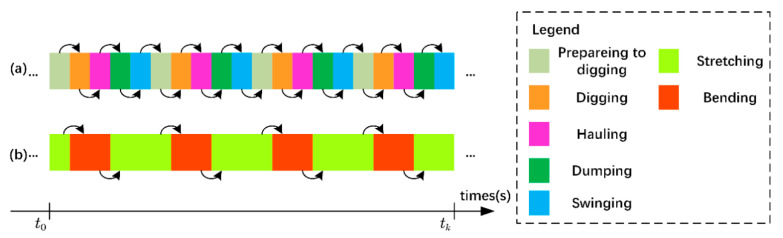
(**a**) Five-atomic-action sequential pattern and (**b**) stretching-bending sequential pattern (SBSP) in video sequence.

The first step to constructing the SBSP is combining atomic actions with a similar visual appearance. We combined the similar visual appearances of the atomic actions “Preparing to dig”, “Dumping”, and “Swinging” into “Stretching”. By the same token, we combined the atomic actions “Digging” and “Hauling” into “Bending”. It was assumed that during time $t_{0}$ to time $t_{k}$ (the time unit is seconds), the earthmoving excavator performed four work cycles in total. Then, the work cycle before combining atomic actions with a similar visual appearance and the atomic actions in the video sequence is shown in the sequential pattern of five atomic actions in Figure 3a, and the work cycle after combining and the atomic actions in the video sequence are shown in the SBSP in Figure 3b. Since the first atomic action of the five-atomic-action sequential pattern is “Preparing to dig”, the first atomic action of the SBSP is “Stretching”. Similarly, the second atomic action of the SBSP is “Bending” according to the operation sequential order. The stretching–bending sequential pattern (SBSP) is constructed by associating “Stretching” and “Bending” in the order of operations, which is obviously simpler than the five atomic action sequential model.

### 2.3. Atomic Action Recognition for SBSP Using SSD

In recent years, many CNN-based object detection models have been proposed, of which single-shot detector (SSD) [13] is one of the most popular object detection models. SSD is based on feedforward neural networks, and is a fast model for object detection using a single deep neural network [13]. It reuses the idea of transforming detection into regression from YOLO [14] to complete target localization and classification in one step, which improves the detection speed; meanwhile, it proposes a default box to improve detection accuracy based on Anchor in Faster R-CNN [15]. Then, in order to find the best detection box, non-maximal suppression is used in SSD to remove the redundant detection boxes and keep the one with the highest confidence to obtain the best position for object detection.

We used a specific SSD model (SSD using MobileNet-V2 [16]) from the Tensorflow object detection API as the atomic actions recognition model. Three parameters were customized to train the model. The first parameter was the *learning rate*, which was used to control the speed of convergence of the model. If the value is too small, the model will become very slow to converge, while if the value is too large, the gradient may oscillate around the minimum value or even fail to converge. The second parameter was the *batch size*, which was used to control the number of samples selected for each training set. The value of *batch size* is related to the GPUs being used, when there are many GPUs with high performance and large memory, a larger value could be set. Such a setting allows more samples to be put into training each time, so that the GPU resources can be fully utilized. On the contrary, a smaller value should be set. The third parameter was *training steps*, which was used to control the number of iterations. If the value of this parameter is too small, the model performance will be poor.

### 2.4. Recognition of Work Cycles

The key to work cycle recognition for earthmoving excavators is the association of atomic actions in the work cycle. Based on the SBSP, we use the intersection over union (IOU) [17], which is commonly used in the study of object detection, to associate the recognized “Stretching” and “Bending” atomic actions in the work cycle according to the actual sequence of operations to recognize the work cycle. The detailed steps are as follows:

(1) The detection box position information and the time information when the n $\left(n\in N^{+}\right)$ atomic action “Stretching” and the first atomic action “Bending” are recognized in the work cycle are stored in the sets $D_{s}$ and $D_{b}$, respectively. The equations for $D_{s}$ and $D_{b}$ are as follows:

(1) $$D_{s}=\left\{d_{s_{1}},d_{s_{2}},\ldots ,d_{s_{i}},\ldots ,d_{s_{n}}\right\},$$

(2) $$D_{b}=\left\{d_{b_{1}}\right\},$$

where $d_{s_{n}}=\left(\left(x_{s_{n}}^{1},y_{s_{n}}^{1},x_{s_{n}}^{2},y_{s_{n}}^{2}\right),t_{s_{n}}\right)$ is the set of information when the “Stretching” atomic action is recognized for the n-th time. $\left(x_{s_{n}}^{1},y_{s_{n}}^{1},x_{s_{n}}^{2},y_{s_{n}}^{2}\right)$ represents the coordinate values of the top-left and bottom-right corners of the atomic action detection box, and $t_{s_{n}}$ records the time information when the atomic action is recognized. Only the information set of the first recognized “Bending” atomic action after “Stretching” is stored in $D_{b}$. When $d_{b_{1}}=\left(\left(x_{b_{1}}^{1},y_{b_{1}}^{1},x_{b_{1}}^{2},y_{b_{1}}^{2}\right),t_{b_{1}}\right)$, $\left(x_{b_{1}}^{1},y_{b_{1}}^{1},x_{b_{1}}^{2},y_{b_{1}}^{2}\right)$ is the coordinate value of the top-left and bottom-right corners of the “Bending” detection box, and $t_{b_{1}}$ records the time information when the atomic action is recognized.

(2) Calculate the IOU value $\sigma_{iou}$ of the first element $d_{s_{1}}$ in $D_{s}$ and element $d_{b_{1}}$ in $D_{b}$ using the IOU. The calculation is shown in Equation (3):

(3) $$\sigma_{iou\left(d_{s_{1}},d_{b_{1}}\right)}=\frac{Area\left(d_{s_{1}}\right)\cap Area\left(d_{b_{1}}\right)}{Area\left(d_{s_{1}}\right)\cup Area\left(d_{b_{1}}\right)},$$

when the value of $\sigma_{iou}$ is greater than 0, the earthmoving excavator has finished one work cycle, which is expressed as $C_{a_{n}}$. In addition, because the location of the earthmoving excavator during the work cycle is usually fixed, a $\sigma_{iou}$ value greater than 0 also determines that the work cycle was completed at the same location.

By subtracting the time when the first element $d_{s_{1}}$ in $D_{s}$ and the element $d_{b_{1}}$ in $D_{b}$ are completed, the time required for “Stretching” to transform into “Bending” in one work cycle is obtained. The calculation is shown in Equation (4):

(4) $$t_{sb}=t_{b_{1}}-t_{s_{1}},$$

(3) Repeat steps (1) and (2) until the recognition of all work cycles of the earthmoving excavator in the video sequence is completed.

(4) Count the number of work cycles. In this paper, only the work cycles of the earth excavator are recognized; therefore, all the work cycles of the earth excavator in the video sequence are set to $C_{a}$. Refer to Equation (5):

(5) $$C_{a}=\left\{c_{a_{1}},c_{a_{2}},\ldots ,c_{a_{i}},\ldots ,c_{a_{n}}\right\},$$

then, the number of work cycles of the earthmoving excavator in this video can be expressed as Equation (6):

(6) $$N_{a}=card\left(C_{a}\right),$$

where $N_{a}$ denotes the number of working cycles of the earth excavator in the video and $card\left(C_{a}\right)$ denotes the number of elements in the set $C_{a}$ of statistics.

It is worth noting that the $t_{sb}$ in Equation (4) is not the time required to complete one work cycle; it is used to recognize abnormal work cycles resulting from driver misoperation. For example, in the case of a five atomic actions sequential pattern, when driver misoperation results in “Digging” after “Dumping”, according to SBSP this is equivalent to the occurrence of “Stretching→Bending→Stretching→Bending” in the work cycle, which means that an abnormal work cycle occurs within a short period of time in one work cycle. Using the proposed work cycle recognition method will inevitably misrecognize one cycle as two cycles. In order to reduce such false recognition, we set the abnormal work cycle filtering threshold as $t_{e}$. This means that in one work cycle of “Stretching” to “Bending”, in the time required for the threshold $t_{e}$ range—i.e., $t_{sb}\subset t_{e}$—the recognized work cycle belongs to the abnormal work cycle and needs to be filtered out from $C_{a}$. The abnormal atomic actions that make up the abnormal work cycle are thus filtered out at the same time.

## 3. Case Study

### 3.1. Experimental Data and Environment

#### 3.1.1. Video Image Dataset

**Figure 4:**
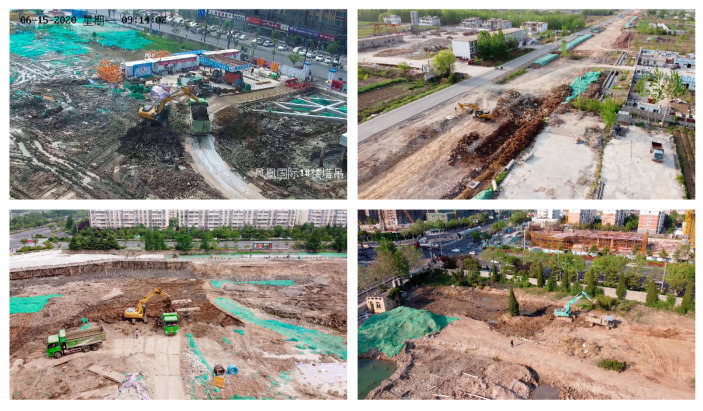
Examples of video data collected from actual earthmoving sites. The two Chinese characters in the first photo in the upper left corner express the recording time of this video (in the upper left corner of the picture: 06-15-2020, Mon, 09:14:02) and the surveillance camera ID (in the lower right corner of the picture: the tower of Phoenix International Building #1).

We obtained surveillance video data from four construction sites. The video data were captured from fixed surveillance cameras installed on four towers in the construction sites. The height of the viewpoint was 20 to 35 m, 40 to 60 m from the earthmoving excavator. The video resolution was set to 1920 × 1080, and the frame rate was set to 25 frames per second (fps). A total of 114,325 frames were captured, representing 76 min and 13 s of working time. Figure 4 shows examples of the collected video data.

We manually selected images containing both “Stretching” and “Bending” atomic actions. Among them, there were 20,645 images with “Stretching” atomic actions and 20,248 images with “Bending” atomic actions. The labelImg software was then used to label the atomic actions in each image (the category and bounding box of the atomic actions in the image), then 6128 and 6008 images, respectively, were randomly selected from them, and the corresponding annotation files of each image were selected as the test image set of the SSD model. The remaining images and the annotation files corresponding to each image were used as the training image set for the SSD model. Our validation sample was a long video 32 min and 40 s in length. The video was manually recognized to have a total of 46 normal work cycles and 24 abnormal work cycles.

#### 3.1.2. Computing Environment

Our method was developed in the Python 3.6 development environment and 64-bit Windows 10 system, with a deep learning framework using Tensorflow 1.14 and video processing using OpenCV 4.4, an open source algorithm library. In terms of hardware configuration, the NVIDA GeForce GTX1080Ti type GPU was used to train the SSD model, and the Intel i5-10600K type CPU was used for atomic action recognition and work cycle recognition.

### 3.2. Experimental Design

To verify the effectiveness of our method, we designed two experiments.

The first was atomic actions recognition based on the SSD model. This experiment first trained the SSD model, and then used the trained SSD model to recognize the atomic actions of the earthmoving excavator in the validation video to examine the performance of the SSD in recognizing two atomic actions. According to our observation in the construction site, the average time taken to complete one work cycle was about 22 s. Therefore, in order to reduce the computational resource consumption, we set to recognize an atomic action every 2 s in the validation video. When training the SSD model, we set the *learning rate* to 0.004, the value of *batch size* to 10, and the *training steps* to $2\times 10^{5}$. For the values of the *learning rate* and *training steps* parameters, we used the default values recommended by the Tensorflow object detection API [18]. For the *batch size*, since a single GPU is used to train the SSD model in this study with small memory, we adjust the *batch size* value from the default value of 24 to a smaller value of 10. This has the advantage of both obtaining a model with stable performance and accelerating the training speed.

The second step was to recognize the work cycles, including two parts of the experiment. ① We used the proposed method to recognize the work cycles in the validation video and examine the performance of the work cycle recognition. ② We used the proposed method for recognizing abnormal work cycles resulting from driver misoperation to recognize abnormal work cycles in the validation video and examine the performance of the abnormal work cycle recognition. In addition, we had to determine the range of the threshold value $t_{e}$. According to our observation in the construction site, the time required to transform “Stretching” to “Bending” in the abnormal work cycle was between 2 and 6 s, so we empirically set the abnormal work cycle filtering threshold $t_{e}$ in the range of [2, 6]—i.e., $2\leq t_{e}\leq 6$. Then, when the time required to transform “Stretching” to “Bending” in one work cycle was within the threshold value $t_{e}$, the work cycle was recognized as an abnormal work cycle and had to be filtered out.

A total of four metrics were used for the evaluation of the above experimental results, which were precision [2,5,7], recall [2,5,7], atomic action average recognition time [3], and single work cycle average recognition time. The results of the first experiment were evaluated using the precision, recall, and atomic action average recognition time. The results of the second experiment were evaluated using precision, recall, and single work cycle average recognition time. The methods for the calculation of the precision and recall are shown in Equations (7) and (8).

(7) $$Precision=\frac{TP}{TP+FP}$$

(8) $$Recall=\frac{TP}{TP+FN}$$

where TP is the number of true positives, which means that the number of atomic actions in which the prediction is true and which are actually true. FP is the number of false positives, which refers to the number of atomic actions that are actually false among the atomic actions predicted to be true. FN is the number of false negatives, which means the number of atomic actions where the actual is true and the prediction is false, including the number of missed detections. The atomic action average recognition time, which in this paper is the average computational time for detecting an earthmoving excavator posture in a single image frame, has units in milliseconds (ms). The single work cycle average recognition time, which is the average time for computing the value of $\sigma_{iou\left(d_{s_{1}},d_{b_{1}}\right)}$ in Section 2.4, is in milliseconds (ms). The atomic action average recognition time and the single work cycle average recognition time can evaluate whether the proposed method can recognize the atomic actions and work cycles of the earthmoving excavator in real time in a surveillance video. The frame rate of the video data used in the experiment is 25 fps, so the atomic action average recognition time and the single work cycle average recognition time should be no higher than 40 ms to achieve real-time recognition.

### 3.3. Results

#### 3.3.1. Recognition Results of the Atomic Action Using SSD Model

There were 6128 images containing the atomic action “Stretching” and 6008 images containing the atomic action “Bending” in the test image set of this paper. The trained SSD model was used to recognize the images containing the atomic action “Stretching” in the test image set, and a total of 6059 images were recognized. The number of images that actually contained the atomic action “Stretching” was 6054. There were five images that misrecognized “Bending” as “Stretching” and 62 images containing “Stretching” that were miss detected. A total of 5988 images containing the atomic action “Bending” were recognized. Among them, 5976 images actually contained the atomic action “Bending”. Twelve images had “Stretching” misrecognized as “Bending”, and 27 images containing “Bending” were misdetected.

As shown in Table 1, the precision of the trained SSD model for the atomic action “Stretching” recognition is 99.92% and the recall is 98.79% in the test image set. There was a 99.80% precision and 99.47% recall for “Bending” atomic action recognition.

Further, we used the trained SSD model to recognize the atomic actions “Stretching” and “Bending” in the validation video. In this validation video, 980 images were detected, of which 393 images actually contained the atomic action “Stretching” and 587 images contained the atomic action “Bending”. The SSD model recognized 377 images containing the atomic action “Stretching”, of which 372 images actually contained the atomic action “Stretching”. The number of images that misrecognized the atomic action “Bending” as “Stretching” was five, and two images that included the atomic action “Stretching” were misdetected. A total of 584 images that contained the atomic action “Bending” were recognized, and 565 of them actually contained the atomic action “Stretching”. The number of images that misrecognized the atomic action “Stretching” as “Bending” was 19, and 17 images that contained the atomic action “Bending” were miss detected.

As shown in Table 2, the precision of the trained SSD model was 98.67% and the recall was 94.66% for the recognition of the atomic action “Stretching” in the validation video. The precision of the “Bending” atomic action recognition was 96.75% and the precisions of the recall was 96.25%. The atomic action average recognition time was 24.49 ms.

**Figure 5:**
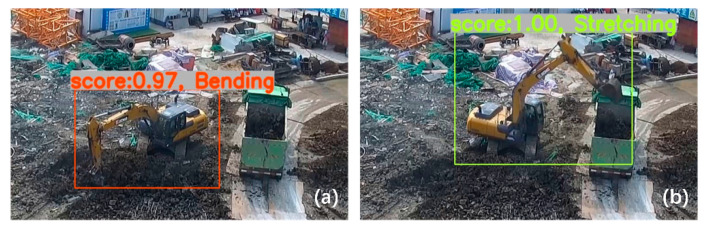
Recognition results for atomic actions: (**a**) bending and (**b**) stretching.

Table 1 and Table 2 show that the performance of our trained SSD model for the test image set and the validation video is acceptable. The Intel i5-10600K type CPU is capable of recognizing atomic actions in real time in the validation video. A possible reason why the SSD model can correctly recognize most of the atomic actions in the validation video is that it benefits from having only two atomic actions in the SBSP with large differences in visual appearance. We selected video screenshots of the recognition results of the two atomic actions in the validation video to show the effectiveness of the SSD model (Figure 5).

#### 3.3.2. Recognition Results of Work Cycles

We recognized 49 normal work cycles in the validation video using the proposed method, of which 45 normal work cycles were correctly recognized. Three abnormal work cycles were misrecognized as normal work cycles and one normal work cycle was misdetected. A total of 23 abnormal work cycles were recognized, of which 21 abnormal work cycles were correctly recognized. Two abnormal work cycles were misrecognized due to atomic action, and zero abnormal work cycles were misdetected.

As shown in Table 3, the proposed method had a 93.75% precision and 97.83% recall for normal work cycle recognition in the validation video. The precision of the abnormal work cycle recognition was 91.30%, and the recall of the method was 87.50%. The single work cycle average recognition time was up to 0.38 ms. The results showed that the proposed method effectively recognized the normal work cycles in the validation video and filtered out most of the abnormal work cycles. Work cycles could be recognized in real time using the Intel i5-10600K type CPU.

**Figure 6:**
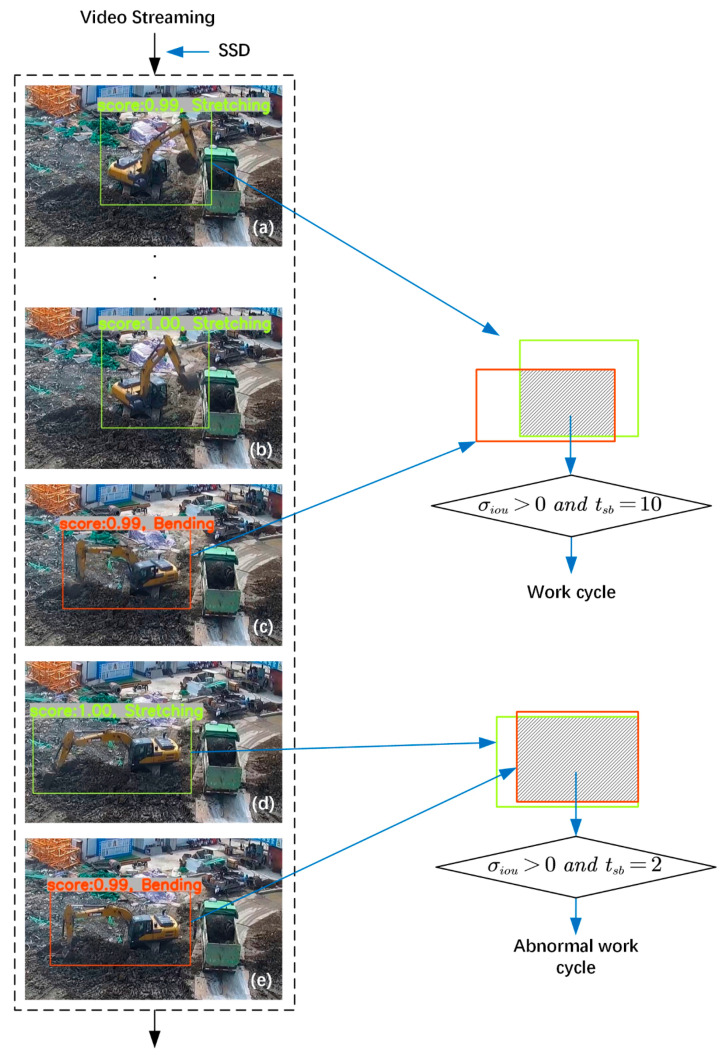
Work cycle recognition. (**a**) “Stretching” was recognized at 09:34:03; (**b**) “Stretching” was recognized at 09:34:11; (**c**) “Bending” was recognized at 09:34:13; (**d**) “Stretching” was recognized at 09:34:15; (**e**) “Bending” was recognized at 09:34:17.

Figure 6 illustrates the process of recognizing a work cycle in the validation video using the method in this paper. Figure 6a–e in the left dashed rectangular box show the results of the recognition of some key atomic actions in this work cycle. Figure 6c shows the driver of the earthmoving excavator executing the “Digging” atomic action prematurely due to misoperation. The transition time for the atomic actions in Figure 6a,c is 10 s. When the driver realizes the error, he re-executes the “Preparing to dig” atomic action in Figure 6d. Then, he executes the “Digging” atomic action again in Figure 6e. The time taken for the atomic action transition in Figure 6d,e is 2 s. Therefore, using the methods in this paper it is possible to obtain values of $\sigma_{iou}$ greater than 0 and $t_{sb}$ values of 10 s for the two atomic actions in Figure 6a,c. Therefore, it can be determined that the group of atomic actions is a normal work cycle. Similarly, using the method described in this paper, we can derive $\sigma_{iou}$ values greater than 0 and $t_{sb}$ values of 2 s for the two atomic actions in Figure 6d,e. Therefore, it can be determined that this group of atomic actions is an abnormal work cycle and can be filtered out. The results of this work cycle recognition show that the normal work cycles and abnormal work cycles of earthmoving excavators can be effectively recognized using the method described in this paper.

## 4. Discussion

### 4.1. Effectiveness of the Proposed Method

We constructed a simplified sequential pattern to recognize the work cycles of earthmoving excavators efficiently in long video sequences. Compared with recognition methods based on temporal sequences [2,10], the sequential pattern can more effectively recognize abnormal work cycles due to driver misoperation, which has been confirmed by the experimental results for work cycle recognition described in this paper (Table 3). Compared with the four atomic actions sequential pattern [11] and five atomic actions sequential pattern, the SBSP constructed in this paper combined atomic actions with similar visual appearances. It was constructed using only two atomic actions, “Stretching” and “Bending”, with obvious visual appearance differences, which reduced the difficulty of atomic action recognition.

Compared with the deep learning-based work cycle recognition method described in the literature [11], our proposed work cycle recognition method is more simple and efficient. Work cycles can be recognized by associating atomic actions in the work cycle using only the IOU. Our method relies more on the performance of the atomic action recognition model. Although many more advanced deep learning-based object detection methods have also emerged in recent years, the detection performance of commonly used SSD models is sufficient to verify the feasibility of our method. According to the experimental results in this paper (Table 2 and Table 3), the performance of our method in the validation video is acceptable. Interpreted from another perspective, our method does not use image information in the recognition of work cycles and can be considered as a simple filtering process at the detection level. The proposed work cycle recognition method, when used on an Intel i5-10600K type CPU, takes an almost negligible time to recognize one work cycle. Meanwhile, the experimental results demonstrate that the method is able to recognize work cycles in long video sequences.

### 4.2. Error Analysis

#### 4.2.1. Error Analysis of Atomic Action Recognition

**Figure 7:**
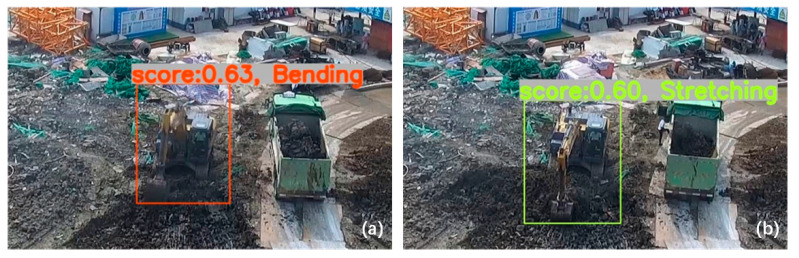
Wrong atomic action recognition. (**a**) “Stretching” is recognized incorrectly as “Bending”; (**b**) “Bending” is recognized incorrectly as “Stretching”.

The experimental results of our trained SSD model for recognizing atomic actions in the validation video show that “Stretching” was misrecognized as “Bending” 19 times, while “Bending” was misrecognized as “Stretching” five times. The main reason for these recognition errors is the self-occlusion of the earthmoving excavator. That is, the rotation of the body at an angle caused self-obscuring. The results for partial misrecognition are shown in Figure 7. The atomic action in Figure 7a is actually “Stretching”, but the SSD model incorrectly recognized it as “Bending”. The atomic action in Figure 7b is actually “Bending”, but the SSD model incorrectly recognized it as “Stretching”. The recognition results in Figure 7 illustrate that when the earthmoving excavator is self-obscuring, it is difficult to recognize its atomic actions using the SSD model.

#### 4.2.2. Error Analysis of Work Cycle Recognition

The results of the work cycle recognition experiment in this paper showed that three abnormal work cycles were incorrectly recognized as normal work cycles. Through our manual analysis, we found that the time required to transform “Stretching” to “Bending” was more than 6 s in all three abnormal work cycles. Although we have found in construction sites that the time required to transform “Stretching” to “Bending” in an abnormal work cycle is usually between 2 and 6 s, there are still a small number of abnormal work cycles with an atomic action transformation times greater than 6 s, which is the main reason for misrecognition.

### 4.3. Threshold Setting

$t_{e}$ and $\sigma_{iou}$ are the two important thresholds of the proposed methods. According to our observation in the construction site, we set the value interval of $t_{e}$ as [2,6]. The results of the work cycle recognition experiments showed that our method correctly recognized 21 abnormal work cycles in the validation video and achieved the filtering of most abnormal work cycles. Table 4 shows the different results of the method in this paper for recognizing abnormal work cycles in the validation video when different $t_{e}$ values were set. According to Table 4, the threshold value $t_{e}$, if set too high, will filter out normal work cycles. If $t_{e}$ is set too low, it will be difficult to filter out most abnormal work cycles.

For $\sigma_{iou}$, we set a larger threshold interval $\left(\sigma_{iou}>0\right)$. This is due to the fact that we mainly recognize the work cycles of single earthmoving excavators and take into account the fact that earthmoving excavators may move slightly during the work cycle. It also indicates that the atomic actions that make up the work cycle are generated by the earthmoving excavator at the same location. If the earthmoving excavator has a slight movement in the work cycle and the $\sigma_{iou}$ value is set too high, the recognition performance of the work cycles will be degraded.

### 4.4. Relationships between the Methods in This Paper and Existing Methods

There are currently four basic types of methods for recognizing the work cycles of earthmoving excavators based on CV. Among them, the proposed method can effectively recognize the abnormal work cycles of earthmoving excavators compared with the recognition based on temporal sequences. When it is necessary to further improve the performance of work cycle recognition for earthmoving excavators, it is also possible to use the proposed method to first recognize the work cycles in the surveillance video in real time, and then manually review the incorrectly recognized work cycles by using the temporal sequence to retrace the atomic actions within the target time interval in the surveillance video. Combining these two methods will not only improve the recognition performance of the work cycles, but also obviously reduce the amount of labor required for manual error checking.

The proposed method inherits and improves the recognition method for work cycles of an earthmoving excavator based on sequential patterns. The major difference between the two methods is that the method in this paper combines atomic actions with a similar visual appearance in the work cycle to the greatest extent. In addition, our work cycle recognition method is simpler and more efficient, providing a new direction for the research on work cycle recognition in earthmoving excavators.

Finally, the existing methods all ignore the impact of real factors, such as driver misoperation, on earthmoving excavator work cycle recognition studies. This enables earthmoving excavators to generate realistic abnormal atomic actions in the surveillance video, which in turn leads to abnormal work cycles. It is the presence of these real factors that makes it difficult to quantitatively compare the existing state-of-the-art method in the literature [11] with our method in the validation video of this paper. The method in the literature [11] is limited in such an application scenario and will find it difficult to correctly recognize the work cycles. This also highlights the advanced character of the method in this paper. As shown by the experimental results of work cycle recognition in this paper, real factors such as driver misoperation have become the main aspects affecting the work cycle recognition performance of earthmoving excavators. This indicates that, in future research, it is important to discover the real factors affecting work cycle recognition based on the actual working conditions and operating specifications of earthmoving excavators in construction sites, and then focus on the real factors to design and develop work cycle recognition methods.

### 4.5. Contributions

The contributions of this paper are as follows:

(1) The existing sequential pattern is simplified and a minimalist SBSP is constructed to reduce the difficulty of atomic action recognition.
(2) A new idea for the recognition of the work cycles of earthmoving excavators is provided. Our method is clearly different from existing methods in that it does not use image information in the work cycle recognition, but rather uses the $\sigma_{iou}$-values of the “Stretching” and “Bending” atomic action detection boxes in the work cycle to achieve work cycle recognition. This method can be considered a simple filtering process at the detection level, making the time spent to recognize a single work cycle on an Intel i5-10600K type CPU negligible.
(3) The real-time recognition of earthmoving excavator work cycles is realized in long video sequences, and abnormal work cycles resulting from driver misoperation are effectively filtered out. The real-time nature of the method in this paper is particularly important in a low-latency construction environment, enabling construction managers on site to calculate the productivity of earthmoving excavators in real time and adjust the construction plan in a timely manner. Moreover, this paper focuses on developing the recognition method of earthmoving excavator work cycles with the consideration of such real factors as driver misoperation. Our method is more suitable to the actual working conditions of earthmoving excavators in construction sites, making it possible to accurately calculate the productivity of earthmoving excavators using computer vision technology.

## 5. Conclusions

This paper focuses on validating an idea to simplify earthmoving excavator sequential patterns to recognize work cycles in long video sequences and filter out abnormal work cycles due to driver misoperation. Based on this idea, this paper first proposed to construct an SBSP. To construct the pattern, we combined the maximum number of visually similar atomic actions in the work cycle of an earthmoving excavator. The original four-atomic-action sequential pattern and our proposed five-atomic-action sequential pattern were further simplified into an SBSP consisting of only two atomic actions. Then, based on the SBSP, we proposed a method to achieve work cycle recognition using two atomic actions in the IOU association work cycle. Based on our observations in the construction site and expert interviews with two experienced drivers, we found the causes of misoperation and proposed a method to filter out abnormal work cycles by using the time required to transform “Stretching” to “Bending” in the abnormal work cycle. The experimental results on the validation video show that the ideas in this paper are feasible.

The method in this paper is different from the existing methods. Compared with the existing four atomic action sequential pattern and our proposed five atomic action sequential pattern, the SBSP greatly simplifies the sequential pattern and reduces the difficulty of atomic action recognition. Compared with the methods based on deep learning used to recognize the work cycles of earthmoving excavators, the work cycle recognition method described in this paper is simpler, and is able to recognize work cycles in long video sequences in real time. However, the accurate recognition of the work cycles of earthmoving excavators also takes into account potential self-obscuring. In addition, our method is only applicable to the recognition of single earthmoving excavator work cycles in surveillance videos; the matter of how to accurately recognize the work cycles of multiple earthmoving excavators in surveillance videos in real time is another important research topic.

## Figures and Tables

**Table 1:** Performance of the single-shot detector model (SSD) for test data.

Atomic Action	Precision (%)	Recall (%)
Stretching	99.92	98.79
Bending	99.80	99.47

**Table 2:** Performance of single-shot detector (SSD) for validation data.

Atomic Action	Precision (%)	Recall (%)	Atomic Action Average Recognition Time (ms)
Stretching	98.67	94.66	24.49
Bending	96.75	96.25	

**Table 3:** Performance of work cycles recognition.

	Precision (%)	Recall (%)	Single Work Cycle Average Recognition Time (ms)
Normal work cycles recognition	93.75	97.83	0.38
Abnormal work cycles recognition	91.30	87.50	

**Table 4:** Effect of $t_{e}$-value on abnormal work cycles recognition.

*t_e_* (s)	Abnormal Work Cycle Recognition	Actual Value
[1,2)	9	24
[2,6]	23	24
(1,12]	40	24

## Data Availability

The data presented in this study are available on request from the corresponding author. The data are not publicly available due to the privacy of construction companies.
